# Neutrophils as Main Players of Immune Response towards Nondegradable Nanoparticles

**DOI:** 10.3390/nano10071273

**Published:** 2020-06-29

**Authors:** Rostyslav Bilyy, Galyna Bila, Oleg Vishchur, Volodymyr Vovk, Martin Herrmann

**Affiliations:** 1 Danylo Halytsky Lviv National Medical University, 79010 Lviv, Ukraine; r.bilyy@gmail.com (R.B.); halynabila@gmail.com (G.B.); vovkvi@yahoo.com (V.V.); 2 Institute of Animal Biology NAAS, 79034 Lviv, Ukraine; vishchur_oleg@ukr.net; 3 Department of Internal Medicine 3—Rheumatology and Immunology, Friedrich Alexander University Erlangen-Nurnberg (FAU), Universitatsklinikum Erlangen, 90154 Erlangen, Germany

**Keywords:** neutrophil, NETs, nanoparticle (nP), nano/microparticles (nµP), plasma membrane, tubular structures

## Abstract

Many nano/microparticles (n/µP), to which our body is exposed, have no physiological way of removal. Our immune system sense these “small particulate objects”, and tries to decrease their harmfulness. Since oxidation, phagocytosis and other methods of degradation do not work with small, chemically resistant, and hydrophobic nanoparticles (nP). This applies to soot from air pollution, nano-diamonds from cosmic impact, polishing and related machines, synthetic polymers, and dietary n/µP. Our body tries to separate these from the surrounding tissue using aggregates from neutrophil extracellular traps (NETs). This effectively works in soft tissues where n/µP are entrapped into granuloma-like structures and isolated. The interactions of hydrophobic nanocrystals with circulating or ductal patrolling neutrophils and the consequent formation of occlusive aggregated NETs (aggNETs) are prone to obstruct capillaries, bile ducts in gallbladder and liver, and many more tubular structures. This may cause serious health problems and often fatality. Here we describe how specific size and surface properties of n/µP can activate neutrophils and lead to aggregation-related pathologies. We discuss “natural” sources of n/µP and those tightly connected to unhealthy diets.

## 1. Introduction

Environmental pollutants (soot, asbestos, cementum, and smog), artificial new materials (microplastic, polymers), naturally-occurring crystals (oxalates, cholesterol), or those generated during metabolism or defense (monosodium urate in gout, hemozoin in malaria, and circulating immune complexes in inflammation) are continuously flooding the human body. A high exposure to n/µP concerns our immune system. All n/µP interact with tissues of our body in a different ways. Recent works demonstrate the growing importance of understanding how n/µP contribute to health, disease development, and prevention [1]. Indeed, phagocytosis, excretion, catabolism, isolation, and immobilization are strategies of the body to handle, or ideally clear, heterogeneous nanoparticles (nP). Several cells orchestrate the response to particulate matter. Epithelial cells constitute physical barriers, mononuclear phagocytes engulf and clear, and granulocytes immobilize and sequester this material. Although this process is usually considered silent, there are conditions causing, often, mild but sometimes also severe pathologies. This is related to conditions when engulfed n/µP cannot be digested, oxidized, dissolved, or excreted. This may be due to inert chemistry (soot, asbestos, cementum, and smog), absence of specific receptors (plastics, soot, and asbestos), high hydrophobicity (cholesterol, hemozoin, nano-diamonds, and polystyrene), or other reasons. In this case, our body will select the strategy of sequestration—isolation of potentially harmful objects in one place and limiting their further contact with body tissues.

In the current manuscript we summarize new findings related to the role of neutrophils upon interaction with hydrophobic, non-degradable nP. We demonstrate the damage of dietary-born nanoparticles (cholesterol and monosodium urate crystals (MSU)) in the hepatobiliary system, effect of soot and related carbon particles in lung tissue, as well as effect of occlusions and obstruction caused by aggregated neutrophil extracellular traps (NETs) in different tissues of the body. In addition, we will discuss the resulting inflammatory responses.

## 2. Materials and Methods 

Studies involving animals, mice, including housing and care, method of euthanasia, and experimental protocols were approved by the Ethical committee of Danylo Halytsky Lviv National Medical University, protocols 20191216/10, 20180226/2, and 20130624/6, all experiments were designed to comply with principles of the 3Rs (replacement, reduction, and refinement). Human tissue samples described in current study were obtained under approval of Ethical committee of Danylo Halytsky Lviv National Medical University, protocol 20180226/02.

Human Jurkat T cells were cultured according to common laboratory practice techniques. When working with human monocytes derived from blood of healthy volunteers we used common best practices and criteria approved at [2,3].

Model for dietary-born nanoparticles. The model of murine NASH [4] using young growing C57BL6/N mice kept under normal ration, high fructose diet (HFD) and high lipid high cholesterol diet (HLHCD), as positive control for NETs formation. The fructose content was 10% in drinking water, roughly corresponding to the content of fructose in soft drinks consumed by humans. At the start of the experiment and, every 14 days, blood samples were collected to measure elastase activity (as described in [4]) and to monitor body weight. After 6 weeks mice gall bladders were surgically removed, and their content was quantitatively transferred into glass slides and examined for the presence of NETs by visualizing DNA with propidium iodine (PI) and neutrophil elastase with specific antibodies using fluorescence microscopy. To prevent gallstone formation animal water was supplemented with 0.0094% w/w of metoprolol [4]. 

The air pouch model was performed by injection of 5 mL of sterile air subcutaneously into the back of Balb/c mice [5]. In two days, an additional 2 mL of sterile air was injected into the pouch. Two days later, 5 mg of n/µP in PBS or PBS only was injected. Then, 24 h later, mice were sacrificed, and the air pouch membranes were analyzed.

## 3. Results

### 3.1. Potential Mechanisms for “Inactivation” of Bio-Resistant Nanoparticles

Polymorphonuclear neutrophilic granulocytes (PMN) and neutrophils are innate immune cells dealing with “particulate objects” found in our body. Neutrophils constitute 40% to 70% of all leukocytes, and their multisegmented nuclei easily allow them to extravasate (leave blood vessels) and patrol surfaces of mucosal linings, ducts, and wounds [6]. Upon contact, neutrophils will phagocytose “particulate material”, produce Reactive Oxygen Species with the aim to inactivate the presumed pathogen. Neutrophils release chemoattractants to recruit further immune cells (more neutrophils, monocytes, and lymphocytes). They are also endowed with a newly discovered weaponry, being the decondensation and externalization of chromatin. They release net-like scaffolds decorated with toxic granular proteins. This process is referred to as formation of neutrophil extracellular traps. Primary or azurophilic granules of neutrophils contain a cocktail of proteinases like neutrophil elastase, cathepsin G, and myeloperoxidase; secondary (specific) granules harbor lysozyme, lactoferrin and collagenase, and tertiary granules contain gelatinase [7]. Just to explain the danger of these proteins: Upon radical oxidation events neutrophil myeloperoxidase catalyzes the production of hypochlorous acid, known as “bleach”. The latter destroys all viable pathogens [8].

The formation of NETs is a heavy weapon used for effective immobilization and destruction of pathogens, for covering wound surfaces [9], and for the prevention of the spread of toxic compounds released during necrosis [10]. Obviously, as in every powerful machinery, it suffers certain drawbacks, which not only include tissue damage during neutrophil hyperactivation (and lung tissue damage during COVID infection is the most discussed example nowadays [11,12]), but also formation of aggregates prone to mechanically obstruct blood vessels [13], and ducts e.g., of exocrine glands [6].

### 3.2. Effect of Nano/Microparticle Size on Its Interaction with Cells or Cell Membranes

**Figure 1:**
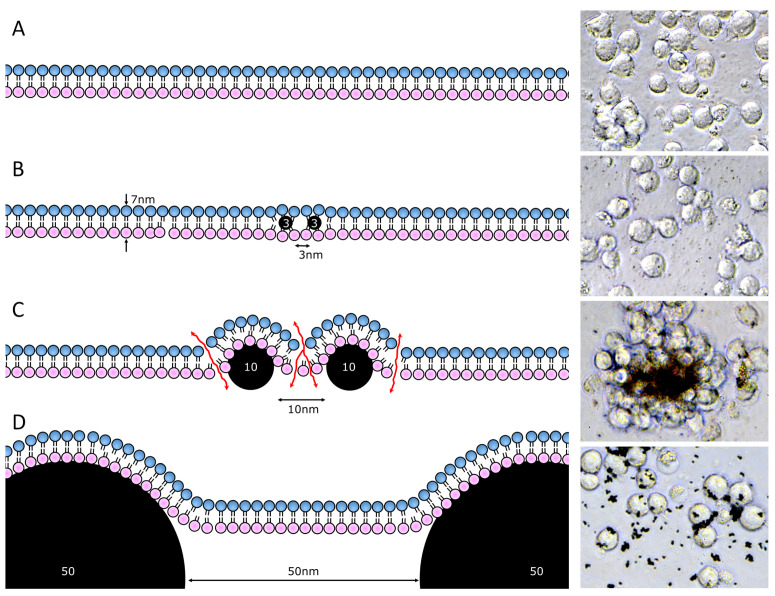
Interaction of lipophilic n/µP with plasma membranes. Schemes demonstrate curvature of lipid bilayer upon interaction with n/µP located on the distance equal to their diameter. Photo inserts demonstrated morphology of Jurkat cells incubated at 2 × 10^6^ per ml overnight with the corresponding n/µP (1 mg/ml of cell suspension). (**A**) intact plasma membrane and untreated cells. (**B**) interaction of plasma membrane and n/µP with diameter smaller than the thickness of PM, e.g., 3 nm nP; cells were treated with fullerenes C60 (van der Waals diameter = 1,1nm). (**C**) interaction with NP whose diameter is between 1× and 5× the thickness of PM, e.g., 10 nm nP, cells were treated with 10nm nano-diamonds. (**D**) interaction with n/µP with diameters more than fivefold the thickness of PM, e.g., 50 nm nP; cells were treated with 200 nm nano-diamonds.

Our recent data suggest that the surface properties and chemical composition has a dramatic effect on its interaction with tissues. In case a n/µP is made of biodegradable material and has a polar surface, it may be phagocytosed and degraded by lysosomal enzymes. In contrast, lipophilic surfaces of n/µP directly interact with lipid bilayer of plasma membranes [14]. The n/µP serve as “detergent” and challenge the tightness of the lipid bilayer. Due to the fixed width of lipid bilayers, being around 7 nm, the interaction with membrane lipids causes a critical membrane curvature determined by the shape of the nP. Based on calculations [15] and experimental data with nano-diamonds of different sizes [14], lipophilic particles with 7 to 40 nm cause membrane leakage for small ions. The nP smaller than 7 nm can easily locate between lipid bilayers without causing membrane damage [16]; nP larger than 50 nm imprint a soft membrane curvature without leakage. Figure 1 depicts the model of lipid membrane interaction with n/µP of different sizes placed apart at the distance equal to their diameter and photo of Jurkat cells after overnight incubation with polar carbon-derived n/µP (fullerenes and nano-diamonds). Lipophilic 10 nm n/µP not only strongly interacted with cell surfaces causing their aggregation/agglutination, but also killed the cells by disrupting the membrane integrity.

### 3.3. Membrane Leakage Can Kill Cells or Induce NETs Formation

**Figure 2:**
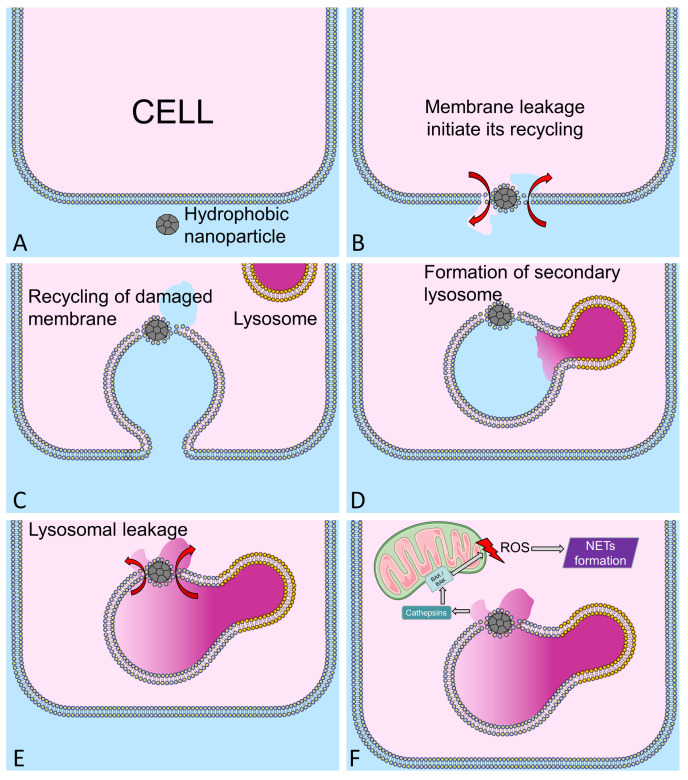
Scheme illustrating the interaction of damaging lipophilic and non-biodegradable nP with plasma membrane. (**A**) nP interact with lipid bilayer causing local leakage (**B**) this initiates events of damage area recycling (**C**) by initiating endocytosis of the membrane area and fusion with lysosomes (**D**). As this the nP cannot be destroyed by lysosomal enzymes, the secondary lysosome containing activated enzymes leaks its content (**E**), in polymorphonuclear neutrophilic granulocytes (PMNs) this events trigger neutrophil extracellular traps (NETs) formation via the activation of mitochondrial ROS production (**F**).

When eukaryotic cell senses plasma membrane leakage, it will immediately start the recycling machinery to determine, isolate, and remove the affected part of plasma membrane and engulf it into endosome [17]. This endosome will consequently fuse with primary lysosomes resulting in the formation of secondary lysosomes. Subsequently the material is recycled. Intact bilayer synthesized and delivered from Golgi complex will then replace the leaky part of the membranes. If lipophilic n/µP were not dissolved by lysosomal enzymes they remain integrated into the lipid bilayer; the leak cannot be remedied. Instead, it will cause lysosomal membrane rupture (Figure 2); toxic lysosomal content enters the cytoplasm. To our estimates, the most damaging size of hydrophobic n/µP is in the range of 10 to 40 nm [14], or roughly 1× to 5× of the diameters of plasma membrane (7–8 nm thick). The lipophilic nature facilitating the integration into the plasma membrane combined with an inability of the cellular machinery to destroy the n/µP will result in cell damage by intrinsic lysosomal enzymes. This will be true for soot, nano-diamonds, nano-plastics, etc. In fact, exposure to polar n/µP with sharp, protruding, lancet, or otherwise irregular but thin shapes within a defined size range will cause membrane damage and induce cellular death. For instance, MSU, asbestos, and CaOx can induce cell death, even if they are substantially larger than 10 nm [18].

The above-mentioned interaction of n/µP and plasma membrane is determined by universal characteristics of chemistry and cell structure. The latter response will differ between cell specifically. From our experience different cancer cells, primary human lymphocytes, macrophages, and RBCs lose cell integrity caused by an increased membrane permeability. Eventually they die after contacting small lipophilic n/µP [16,19,20,21,22]. Cells able to phagocytose will engulf n/µP before death, like murine macrophages J774.2, and human monocyte-derived macrophages. Human epithelia-derived HeLa cells formed big vacuoles before cell death [14]. 

In neutrophils, specialized in immobilization and degradation of particulate matter, the engulfment of hydrophobic n/µP and the resulting lysosomal leakage activates further molecular cascades resulting in generation of ROS and initiation of NETs formation. It has been demonstrated that in acute kidney inflammation (AKI), crystal-induced necroptosis occurs, involving mitochondria permeability transition [23]. After secondary lysosome leaks, cathepsins enters the cytoplasm, and the cells produces ROS. This is accompanied by mitochondrial swelling, loss of mitochondrial outer membrane potential, and loss of cristae. Mitochondria permeability transition pore are formed, which in turn induces even more ROS production self-catalyzing the process [23] (Figure 2F). 

### 3.4. Dietary-Born Nano/Microparticle and NETs Formation

NETs display a protective potential in our body. They immobilize and destruct fungal hyphae [24]; they isolate areas of necrosis and sequester dead tissues [10], or immobilize microcrystals of monosodium urate, the trigger of gout [25]. They sterically isolate damaging particles and attract further immune cells to stimulate the fight with pathogens. In the late phase of infection, proteases released from neutrophils’ granules tend to cleave pro-inflammatory cytokines and limit the spread of inflammation [25]. 

However, when NETs formation is exaggerated, and this may occur with chronic exposure to certain nP, excessive NETs formation and aggregation poses a threat to the host. NETs may aggregate and, consequently, sterically block ducts and vessels. Excessive NET formation reportedly contributed to the occlusion of blood vessels [26], and pancreatic ducts [6]. They initiate formation and growth of gallstone often associated with the occlusion of the hepatobiliary ducts [4].

#### 3.4.1. Monosodium Urate Nanocrystals

Uric acid is one of the major byproducts of the human catabolism, also being a strong antioxidant. The normal concentration range of uric acid (or hydrogen urate ion) in human blood is 25 to 80 mg/L for males and 15 to 60 mg/L for females [27]; the solubility of uric acid is 60 mg/L at 20 °C. The cellular concentration of uric acid can even be higher; since the intercellular milieu is low in sodium no uric acid salts form and precipitate. After uric acid binds sodium the monosodium urate crystals forms, with much lower solubility, and it tends to precipitate into needle-like crystals [28], the causative agent of gout. For centuries gout was connected with excessive uptake of meat, fat, and other dietary products. Despite MSU needles being micrometers long, they possess sharp edges. PMNs effectively engulf MSU and trigger NET formation [29]. The pyrogenic action of MSU is mediated through their uptake by monocytes, the formation of NALP3 inflammasomes and the release of IL-1β [30]. IL-1β alarms and activates neutrophils together with further immune cells.

Neutrophils directly ingest MSU crystals and initiate inflammasome-dependent NET-formation [31,32]. This sequesters the MSU crystals inside aggregated NETs and provoked self-limiting inflammation finally resolving inflammatory gout attacks.

#### 3.4.2. Cholesterol Nanocrystals

Gallstones are mainly made of cholesterol, bile acids and calcium deposits and can easily rich few centimeters in diameter. Cholesterol crystals, the first known lipid crystals, have been discovered in Lviv University more than 150 years ago [33]; they are common in human bodies. They can spontaneously and physiologically appear in gallbladder *luminae* [34]. Neutrophils, on the other hand, are patrolling bile ducts, since these connect the vital liver with the intestine. The latter is full of bacteria (some animals does not even have bile ducts, and release bile directly into the intestine). Hydrophobic cholesterol crystal can cause cellular death [18]. Cholesterol crystals in gallbladder luminae can cause NET formation and, consequently, lead to a luminar occlusion [4]. An autocatalytic vicious circle develops; the more NETs you have, the bigger grows the stone. In the hypersaturated bile the aggregated NETs get continuously impregnated with calcium and other salts. Our recent finding reported that NETs are initiating gall stone formation and growth [4]. Employing an animal model with lithogenic diet, we directly demonstrated how NETs entrap cholesterol crystals (Figure 4). The NET-borne DNA clumped cholesterol nanocrystals [35], served as glue and was present on the surfaces of the gallstones. Indeed, the cross-section of the gallstones of mice fed a high-fat diet showed onion-skin-like morphologies and high neutrophil elastase activities. The latter enzyme is rather stable; nevertheless, the younger parts of the stones were endowed with the highest elastase activities [4]. Active inhibition of NETs formation significantly reduced development and growth of the gallstones [4].

#### 3.4.3. Fructose

Fructose is a highly soluble compound. Unfortunately, it is a cheap source of sugar in corn syrup, that is extensively used in soft drinks. Recently fructose has been linked to hepatopathology [36]; non-alcoholic steatohepatosis (NASH) being the most important. The content of fructose in some soft drinks can reach 65 g/L [37] or even more, making it a major metabolic player. Fructo-phosphokinase, converts fructose into fructose-1-phosphate and consumes 1 ATP molecule. Unfortunately, the hepatic fructo-phosphokinase has no negative feedback regulation. Consequently, all fructose molecules available in liver cells are converted into fructose-1-phospahate using the corresponding amounts of ATP. During evolution a negative control of the enzyme was not needed since fructose was scarce even in the summer. This was true even few decades ago, before modern nutritional changes the availability. When consumed in a dozen of grams quantities, fructose will quickly deplete hepatic ATP stores causing metabolic starvation and death of liver cells. Fructose intake was, for a long time, connected with increased purine metabolism and increased uric acid production [38] and recently with established link to many resulting disorders [39]. Thus, high fructose diet will cause increased production of MSU nanocrystals.

**Figure 3:**
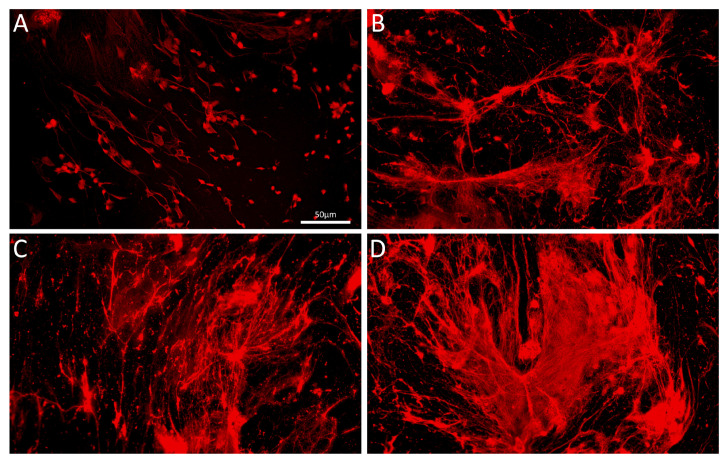
Gallbladder content, isolated from mice fed high-fructose diet for 4 weeks (**A**,**B**) and 6 weeks (**C**,**D**). DNA was stained with propidium iodide (red). Images A to D represent progressive stages of NETs formation endowed with a high tendency to aggregate; magnification is constant.

In the experimental murine model for NASH we employed the C57BL/6N strain. When fed high lipid high cholesterol diet, the mice developed nano-alcoholic fatty liver disease and NASH within 4 and 6 weeks, respectively. The symptoms were accompanied by an increased NETs formation in the gall bladder and increased neutrophil elastase activity in the sera. Changing the dietary conditions from high-cholesterol to high-fructose (10% fructose in drinking water corresponding to extensive consumption of soft drinks), we have observed similarly increased levels of serum elastase activity as well as strong formation of NETs inside the gallbladder [40]. Blocking diapedesis of PMNs by metoprolol effectively decreased amount and size of gallstones under high lipid high-cholesterol content [4], but also decreased serum elastase activity and NETs in gall bladders under either high fructose or high cholesterol diets (Supplementary Materials Figure S1). Fluorescence microscopy revealed that upon formation in the gallbladder NETs tend to spread and form aggregated NETs (Figure 3). These may contribute to the occlusion of the biliary duct system. Surprisingly, the combination of high-fructose and high cholesterol diet did not contribute to further increase in elastase activity suggesting some upper threshold amount of NETs that can be formed.

Current lifestyle greatly promotes the junk food, which will be inevitably rich in cholesterol (due to frying stuff) and fructose (mainly due to soft drinks). It is prone to induce increased levels of either lipophilic n/µP or sharp n/µP both formed in the gastrointestinal tract and the hepatobiliary ducts. These particles stimulate the formation of NETs with a high tendency to aggregate.

### 3.5. Consequences of Nano/Microparticle Aggregation in Tissues and Ducts/Vessels

NETs allow our body to survive under the constant load of potentially dangerous n/µP and to sequester and isolate from surrounding tissues those that cannot be safely degraded. It is fair to assume, that any accumulation of foreign material in the body carries risk for the health. In this context, NETs can worsen or improve the situation.

The initially formed NETs derived from sentinel cells and the content of neutrophil granules will attract further neutrophils to the area of contact with the nP. When they reach a critical concentration, enough to form aggregated NETs, the latter will shield the n/µP from viable areas of the tissue. The damaged cells will then cause local inflammation. 

**Figure 4:**
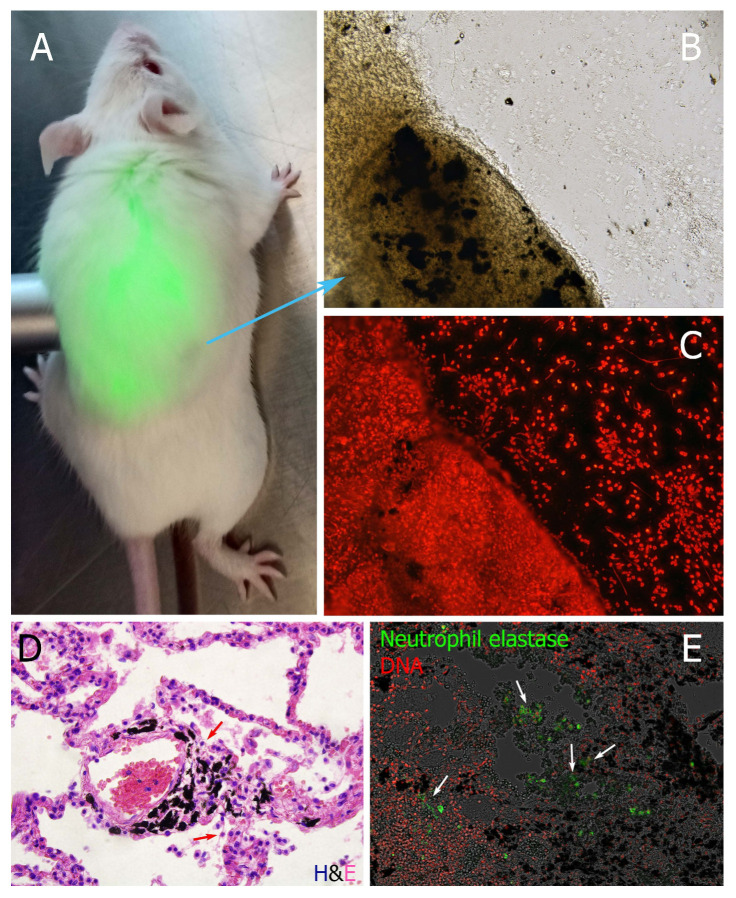
Sequestration of soot n/µP by NETs. (**A**) mice injected with soot suspension into the air pouch after 24h demonstrate accumulation of all black material in one spot. Histology of this material (**B**) and fluorescence microscopy (**C**) revealed that neutrophils externalized DNA and entrapped the soot, objective: 10×. (**D)** lung tissue of light smoker demonstrating concentration of dust materials in some specific areas of lung tissue. Externalized DNA and neutrophils elastase surrounded these areas (**E**). (**D**,**E**) objective is 40× 0.75NA.

**Figure 5:**
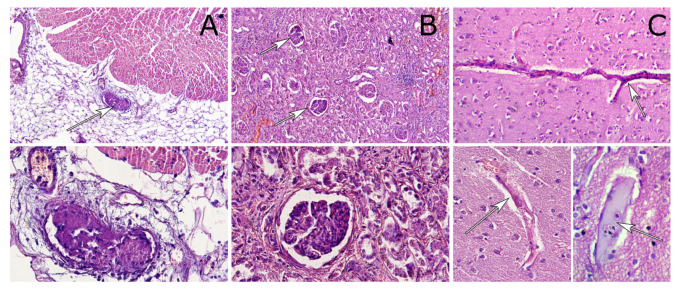
Human autopsy samples demonstrated basophilic DNA-rich clots in heart (**A**), kidney (**B**), and brain (**C**) tissues. H&E staining, Upper row—objective 10×, lower row—objective 40×.

Formation of aggregated NETs will be accompanied by the release of DNA-bound cytokine-degrading proteases [25] as well as enzymes degrading circulating immune complexes [41]. This makes the inflammation self-limiting. Indeed, even sterile n/µP often cause self-limiting inflammation [42]. The granuloma-like structures formed under certain conditions will sequester nanomaterial and limit its spread in the body (Figure 4 and Figure 5).

#### 3.5.1. Beneficial Role of Nano/Microparticle Aggregation

The beneficial role of n/µP aggregation is definitely in minimizing the area of contacting cells, which can be damaged, for example with soot nP. In patients with tuberculosis NETs effectively surround granulomas in the peritoneal cavity. In this way neutrophils collect and sequester pathogenic bacteria in a single area. This formed a shield and limited pathogen spread. Similar patterns have been observed when carbon particles were inhaled by humans. Contrary to the expected uniform distribution the dust particles were concentrated in few areas (Figure 4D,E). Many of them had been engulfed by alveolar macrophages. Importantly, extracellular DNA and externalized neutrophils elastase surrounded the aggregates [21,22]. These conditions can be reproduced when mice with induced air pouches were injected with soot. Soon after injection, soot was covering all the surface of air pouch. However, after 24h it was accumulated in a single spot (Figure 4). Analyses by histology revealed abundant neutrophils covering and entrapping soot particles. This mechanism limits the inflammation caused by carbon-induced cell death to only a few areas of the lung, keeping the surrounding tissues alive and functional.

Using adjuvants, n/µP are intentionally injected into the organism, usually into soft tissues. NETs contribute to the controlled immune boost, needed to generate a sufficient immune response which is observed when vaccines are co-injected with particulate matter like aluminum oxide or hydroxide [43]. The latter are typical non-degradable nP. Aluminum oxide nanowires with a thickness of 20 nm and a length 20–60 µm stimulated NET formation and enhanced the adjuvant-boosting properties. This way of application increased antibody titers 16-fold when compared to amorphous aluminum oxide powder [44].

To track the ability of neutrophils to concentrate n/µP we have injected fluorescent nano-diamonds (fluorescent due to induced surface defects) intraperitoneally or intratracheally into mice. Then we followed distribution kinetics using in vivo fluorescent analysis of the whole animal. The signals gradually concentrated in limited areas in both abdominal cavities and lungs, correspondingly. After 5 days we observed diamond n/µP to be concentrated within granulomas, covered with DNA-rich material (NETs) (Figure 5A). In the lungs n/µP were concentrated near bronchioli associated with abundant extravasation of neutrophils. 

#### 3.5.2. Detrimental Effect of Nanoparticle-NET Aggregates

Aggregated NETs have a physical consistency of bubble gum; they are sticky, elastic, and insoluble, but highly absorptive due to the negative charge of the DNA. The most dangerous places in the bodies where these aggregates are prone to cause life-threatening conditions are vessels, ducts, and air spaces.

In the circulation humans harbor an NET-resolving machinery, consisting of circulating DNase1 and DNase1L3 [13]. The enzymes effectively prevent NET formation in the circulation. Failure of both enzymes, e.g., by overwhelming NET accumulation caused vascular occlusion and small vessel thrombosis. The smallest vessels of the human body can be found in lungs and brain. They load blood with oxygen in the former and supply oxygen to the latter. From a morphological point of view, these clots are basophilic in nature, which is opposite to the canonical eosinophilic fibrinous clots. In autopsy samples of individuals with multiple organ failure related to sepsis and systemic inflammatory response syndrome (SIRS), we observed clots, exposing PMN markers and externalized DNA in distant, presumably not directly affected organs, like lungs, brain, and kidneys (where thin capillaries are first clotted) (Figure 5).

## 4. Discussion

Presented data indicates the importance of the control of NET formation during systemic inflammation. Recent findings suggest possible NETs involvement in severe malaria [45], with hemozoin being insoluble crystalline n/µP triggering the neutrophil activation [46]. Artificial injection of n/µP into the bloodstream of mice induced death within few minutes due to ischemic damage of brain (article in preparation). 

**Figure 6:**
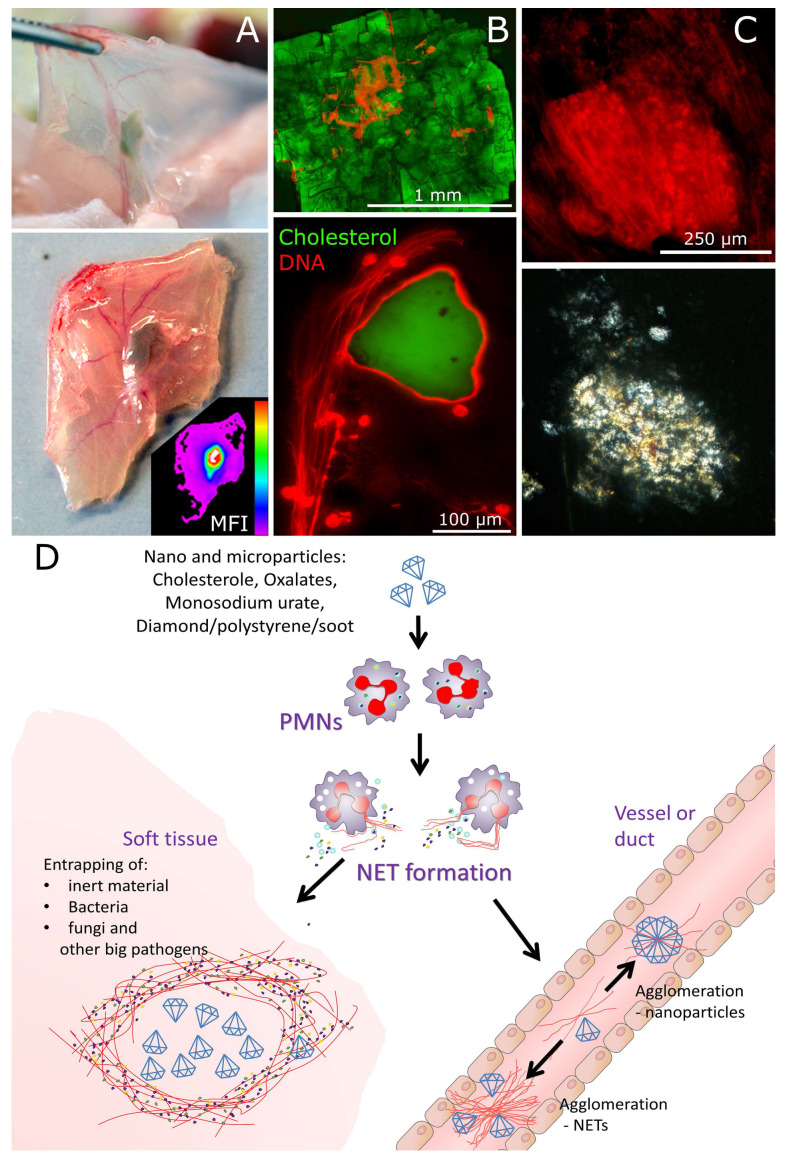
NETs are converting n/µP to macroaggregates. (**A**) granuloma-like structure, positive with DNA formed around fluorescent nano-diamonds injected into the abdominal cavity of mice, insert demonstrated measure MFI using InVivo Extreme fluorescent imager. Tissue was analyzed 5 days post-injection. (**B**) DNA fibers can be found inside the macroscopic cholesterol crystals, NETs are aggregating cholesterol nanocrystals resulting in the formation of macroaggregates in the ducts and vessels. (**C**) upon injection of cholesterol to air pouch cavities we observed covering of big cholesterol aggregates with NETS (fluorescent and polarizing microscopies). (**D**) schematic representation of possible events leading to either sequestration of n/µP in soft tissues (left), or formation of aggregates of NETs of n/µP in ducts and vessels (right).

As reported previously [4], in the bile ducts NETs serve as a nidus of gallstone formation. The NETs directly glue and aggregate cholesterol nanocrystals (Figure 6B). Eventually this results in formation of bigger aggregates, their calcification, and, finally, the formation of gallstones. These may occupy the whole space of the gallbladder or, even worse, block the hepatobiliary duct system. This may also apply for further ductal systems of the human body. 

On the surfaces of body cavities NETs tend to aggregate and sequester n/µP and cluster them in few spots. However, under flow conditions NETs upon contact with n/µP can either self-aggregate (e.g., vascular clots) or, like in gallstones, aggregate the n/µP and support the growth of the aggregates. If this happens in the microvasculature or in narrow ducts (e.g., intrahepatic biliary ducts) this results in a permanent occlusion and mechanical damage of the organ. The importance of the site of NETs formation was demonstrated by the injection of cholesterol crystals into murine air pouches. In ducts NETs tend to agglomerate small cholesterol particles and were seen inside the crystals. In contrast, in the relatively stable environment of the air pouch NETs covered the whole cholesterol crystals (Figure 6C). Thus, not only the nature of nP, but also the place of its location in the body will differ in their response. These sequences are schematically shown in Figure 6D.

Polar n/µP possess the permanent danger upon their contact with body tissues and our body utilize the developed protection mechanism by sequestering n/µP using neutrophils and neutrophil extracellular traps. This can have beneficial consequences resulting in the removal of the contact with dangerous agent with living cells and strong local immune response. Polar n/µP can also be detrimental as big nP-aggNET clusters can block or occlude vessels or ducts causing hypoxia and severe damage to the tissue and to the organism as a whole, respectively. Besides, phagocytosis of crystals such as silica, asbestos, and monosodium urate by neutrophils/macrophages may cause lysosomal damage thereby activating inflammasome complex and exacerbating inflammation and this complex question needs further investigation.

## Supplementary Materials

The following are available online at https://www.mdpi.com/2079-4991/10/7/1273/s1, Figure S1: Elastase activity in blood serum of mice under high-fat high-cholesterol diet, high-fructose diet, and under the influence of blocker of PMN extravasation.
