# Supplementary material for: Neutrophils as Main Players of Immune Response towards Nondegradable Nanoparticles
Source: Nanomaterials (Basel). 2020 Jun 29;10(7):1273. doi: 10.3390/nano10071273 (PMC7408411; doi:10.3390/nano10071273)
Supplement: Supplementary file 1 [file nanomaterials-10-01273-s001.pdf]

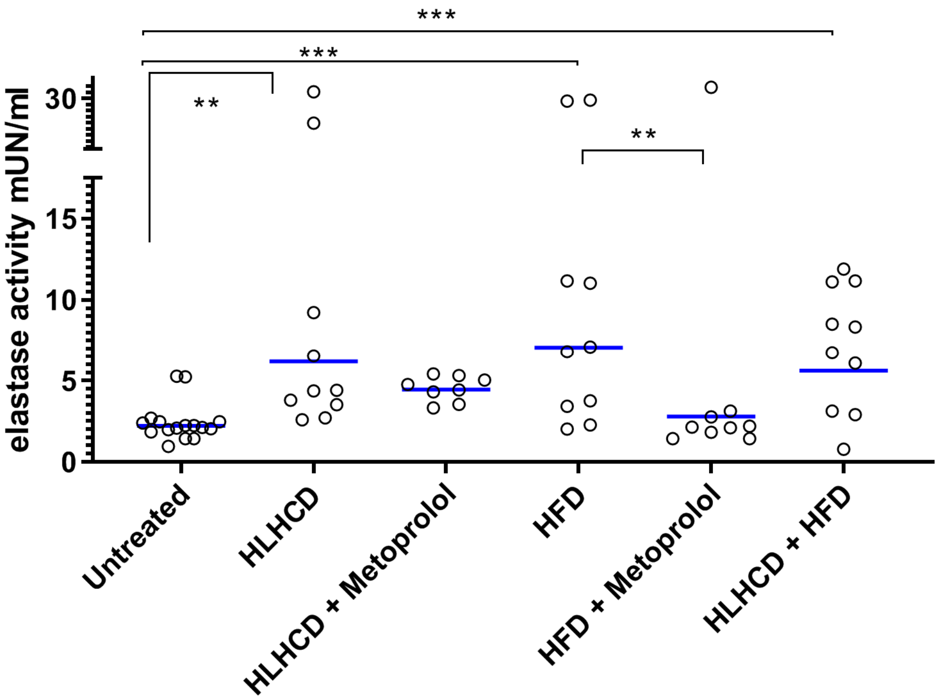

Figure S**1.** Elastase activity in blood serum of mice under high-fat high-cholesterol diet, high-fructose diet, and under the influence of blocker of PMN extravasation.
